# Smart Tree: An Architectural, Greening and ICT Multidisciplinary Approach to Smart Campus Environments

**DOI:** 10.3390/s21217202

**Published:** 2021-10-29

**Authors:** Sergio Fortes, Noelia Hidalgo-Triana, Juan-Manuel Sánchez-la-Chica, María-Luz García-Ceballos, Juan Cantizani-Estepa, Andrés-Vicente Pérez-Latorre, Eduardo Baena, Andrés Pineda, Jorge Barrios-Corpa, Alberto García-Marín

**Affiliations:** 1 Instituto de Telecomunicación (TELMA), Universidad de Málaga, CEI Andalucía TECH E.T.S. Ingeniería de Telecomunicación, Bulevar Louis Pasteur 35, 29010 Málaga, Spain; jce@ic.uma.es (J.C.-E.); ebm@ic.uma.es (E.B.); apg@ic.uma.es (A.P.); 2 Department of Botany and Plant Physiology (Botany Area), Faculty of Science, University of Málaga, CEI Andalucía TECH, Bulevar Louis Pasteur 31, 29010 Málaga, Spain; nhidalgo@uma.es (N.H.-T.); avperez@uma.es (A.-V.P.-L.); 3 Departamento de Arte y Arquitectura, Universidad de Málaga, CEI Andalucía TECH, Plaza del Ejido s/n, 29013 Málaga, Spain; mane@uma.es (J.-M.S.-l.-C.); jbarrios@uma.es (J.B.-C.); albertogm@uma.es (A.G.-M.); 4 Departamento de Expresión Gráfica, Diseño y Proyectos, Universidad de Málaga, CEI Andalucía TECH, C/Doctor Ortiz Ramos s/n, 29071 Málaga, Spain; mlgarcia@uma.es

**Keywords:** smart campus, smart city, urban greening, climate change mitigation, environmental monitoring, circular economy, friendly outdoor spaces

## Abstract

At present, climate change, pollution, and uncontrolled urbanism threaten not only natural ecosystems, but also the urban environment. Approaches to mitigate these challenges and able to provide an alternative for the use of the space are deemed to be multidisciplinary, combining architecture, vegetation integration, circular economy and information and communications technologies (ICT). University campuses are a key scenario to evaluate such solutions as their student and research community is intrinsically willing to support these experiences and provide a wide knowledge on the fields necessary for their design and implementation. However, the creation of areas combining usability and sustainability is commonly lacking a multidisciplinary approach combining all these different perspectives. Hence, the present work aims to overcome this limitation by the development of a novel integrated approach for campus spaces for co-working and leisure, namely a “Smart Tree”, where novel architecture, furniture design, flora integration, environmental sensoring and communications join together. To this end, a survey of the literature is provided, covering related approaches as well as general principles behind them. From this, the general requirements and constraints for the development of the Smart Tree area are identified, establishing the main interactions between the architecture, greening and ICT perspectives. Such requirements guide the proposed system design and implementation, whose impact on the environment is analyzed. Finally, the research challenges and lessons learned for their development are identified in order to support future works.

## 1. Introduction

In the United Nations Millennium Declaration [1], a set of 17 Sustainable Developmment Goals (SDGs) were established, including ensuring environmental sustainability, highlighting the reduction of carbon dioxide emissions (SDG 13), both of a direct industrial type and those generated by transport [2].

Additionally, among the main risks for the Planet [3], taking into account their degree of probability and the level of impact on society, episodes of extreme weather and climate change are the most relevant [4]. Both cases represent a consolidation of the level of concern with which environmental risks are perceived.

Moreover, the progressive urban growth, the number of students at the Universities and the lack of open-air co-working spaces has led the Universities to set up spaces, making its campus university a smarter, more comfortable and sustainable campus. In response to this need, Smart solutions become an experimental laboratory for Smart Cities. In a small space, they provide the necessary conditions for sustainability, nature and new technologies to combine, offering results that can be extrapolated to other areas or scales.

Focusing on the pandemic that we are suffering, the scientific evidence that the transmission of the SARS-CoV-2 virus decreases markedly in outdoor spaces has led notable researchers to recommend that professional activities be transferred to the open air [5]. These circumstances, which may be repeated in the future, make it necessary to implement open-air work spaces in locations with Mediterranean climate such as the city of Malaga (Spain).

In the current environmental and climatic context, the mitigation and adaptation obtained of urban greening with plants [6,7,8,9] are essential and aligned with SDG 13. Urban greening is the act of incorporating or promoting nature in urban areas through the recovery of the native flora (adapted plants) and fauna in an acceptable and sustainable manner in structures made by humans [6,7].

Among the beneficial effects for humans of the urban greening can be mentioned: (1) mitigation of maximum and minimum temperatures [10,11,12], (2) maintenance of shade and humidity [10,13,14], (3) quality improvement of the air, (4) carbon retention reducing the greenhouse effect by providing oxygen in the unbreathable environments of the urban cores [15,16], (5) barrier against pollution and biotic/abiotic particles [17], (6) screen effect against noise [18], (7) food material for pollinating insects and finally, (8) the introduction of biodiversity in artificial human environments improve the general lansdcape, with possible uses in environmental education of all these effects [19,20,21,22,23].

Responsible production and consumption is SDG 12, which aims to do more with less, increasing resource efficiency and reducing environmental degradation. It also aims to decouple economic development from the voracious consumption of natural resources [2]. To achieve this SDG, the transformation of the current global economy is a priority; we must move from the traditional linear business model to a circular economic model based on the reduction of environmental impacts on the environment and the valuation of raw materials. To obtain the valuation of raw materials, the concept of waste must be forgotten and products must be reintegrated back into the production process once their first useful life is over.

Establishing connections between the design and management of resources makes it possible to identify strategies that, from product engineering, will help to apply the principles of the circular economy.

One of the key aspects to achieve sustainable areas is the use of information and communications technologies (ICT) involving sensors, communications and data processing. Here, the IoT (Internet of Things) paradigm is currently one of the fields of computing whose development is having the greatest impact on society. According to Cisco IBSG (Internet Business Solutions Group) the IoT emerged between 2008 and 2009 [24], years in which the number of connected devices exceeded the number of people on Earth. In essence, the objective of this paradigm is to allow objects to be connected to the Internet, in order to have control over them, to know their status or their environment, as well as other more complex applications. Thus, in the field of IoT there is a need to “sensorize” a huge number of devices. Subsequently, the data collected by these sensors are sent to other network elements, which allow data processing, or simply display the collected information.

Thus, IoT represents the first real evolution of the Internet, where information is seamlessly integrated with the real world of things. Many advances are under adoption thanks to this field, such as the smart city concept. A smart city is essentially a city equipped with a management system where all its devices are interconnected. This capacity supported by new technologies allows an efficient use of municipal services and urban environment conditioning such as public transport, environmental conditions, optimization of energy resources, waste control, etc. [25]. Thus, a smart city should be able to detect the needs of its inhabitants and provide a quick and efficient response, as well as know its status. Moreover, in problems as important today as environmental impact, IoT and smart city approaches are key [26].

As part of the Smart City paradigm, and a key scenario for it is the concept of Smart Campus, born from the application of the Smart City concept to the area, services and environment of the university campuses. These are a extremely relevant use cases, as they typically contain very large spaces, with multiple buildings, green areas, services (water, electricity…), huge mobility and a “population” prone to accept and collaborate in the deployment and evaluation of new technologies [25]. Here, the research community as well as students can help the design, implementation, operation and assessment of the different initiatives providing and invaluable environment to their test, to contribute to the sustainability and well-being of the campus as a first-step before bringing that solutions to larger areas, such as complete cities.

The indicated previous smart campus and smart city works establish some of the foundations for a smart campus in terms of open data collection, and a natural and sustainable campus in terms of greening outdoor spaces. However, they commonly focus on just one aspect of such development. e.g., ICT. In this way, there is a shortage of works integrating multiple disciplines and implementing novel concepts such as “environmental comfort” (in relation to the improvement of well-being in outdoor work environments) and “circular architecture” (concerning to minimize environmental impacts and maximize economic value through new processes that reformulate detection, cataloging and redesign from materials destined for waste).

As is the case in many of the new spaces in our cities created in the eighties of the last century, the university campus suffers the consequences of a design in which the car played an excessive role. With the intention of reactivating this environment and provide a key testcase for the Smart Campus paradigm, the present work defines the University of Malaga (UMA) “Smart Tree”, a sensing and smart environment which combines novel architectural, greening, furniture and ICT approaches to provide a sustainable and “smart” area for leisure, study and co-working in the campuses.

In this context, the present work provides a detailed description on how the defined Smart Tree development aimed at addressing the problems that affect university campus students every day in order to promote communication, relationships, environmental awareness and social participation; the integration of vegetation that improve the urban environment; and the integration of ICTs. All this in such a way that life in the university environment is more friendly and comfortable, as an example that can be scaled to other urban areas.

The main contributions of the present work are in the the definition, development and implementation of an innovative small outdoor area combining a novel architectural design guided by the circular economy paradigm, urban greening and ICT sensoring. Such environment is born as way to test different technologies and approaches as well as to serve as a place for leisure, teaching and study with a strong sustainability target as well as for the study of vegetation and to contribute to the environment and to the biodiversity of the Campus. Moreover, the main state of the art, lessons learned and research challenges for this type of approaches are defined and analyzed.

In this way, the present article describes the main integrated approach of the Smart Tree initiative. Firstly, Section 2 analyzes the key related works and bibliography for the different perspectives of the project. From these, Section 3 presents the Smart Tree requirements and the high-level proposed system. Section 4 focuses on the architectural aspects of the design, while Section 5 describes the greening approach and Section 6 presents the sensing and telecommunications of the system. From the design and implementation of the system, the lessons learned and the open research challenges are gathered as compiled in Section 7. Finally, Section 8 presents the conclusions and outlook of the work.

## 2. Related Work

The Málaga University Campus, tries to learn from the American campuses in terms of effectiveness and efficiency [27], where green areas and pleasant recreational spaces dominate the exterior of the buildings. Campuses that optimize the range of use of the spaces by sharing, between the different schools, all those functions susceptible to common use.

The University of Malaga has a main campus (Campus of Teatinos) with a total area of more than 880,000 m^2^ where the total of green areas available are about 160,000 m^2^ (personal observation) which have to be shared by more than 40,000 users. This represents approximately 4 m^2^ of green areas/person (personal observation), less than the 10 squared meters recommended according to World Health Organization (WHO).

To improve conditions on university campuses, other universities in Spain have taken some first steps in this regard, such as the University of Zaragoza [28], which has a spatial and geographic information system on the campus that allows consulting the inventory of facilities and classrooms available at all times, or the University of Alicante [29], which has a management system for the mobility of vehicles that come to the campus and monitor the use of the car parks.

In contrast to the strategies mentioned above, Smart Tree is a project that combines architecture, botany and information technologies to regenerate a disused space at the University of Málaga. This small intervention is intended to be repeated in the future in other areas of the university campus generating a network of green spaces connected to each other, forming a “smart forest” that transforms the public space of this part of the city.

This way of regenerating public spaces emerged at the beginning of the 21st century through different interventions that combine citizen participation, the creation of interactive spaces, climate design and architecture. The architecture studio “Ecosistema Urbano” has developed different interventions, among which the “Eco-Bulevar—Air Tree, 2004” [30] in Madrid stands out, through which artificial trees are generated to support vegetation, energy capture and connectivity systems. The objective of the “Tree of Air” was to generate a comfortable public space in the neighborhood of Vallecas.

The Smart Tree concept also has relation with other interventions of the architecture studio Ecosistema Urbano, such as “Air Tree—Shanghai 2010” [31], “Cuenca Red—2010” [32], whose purpose is also to regenerate deteriorated public spaces. Both projects aim to improve public spaces through architecture and citizen participation, incorporating sensor systems that can be consulted through computer applications available on cell phones. Both projects are also intended to be replicated in different locations to form a network of connected spaces.

Other architectural firms such as Elii Architects have developed urban regeneration projects such as the “P.N.S General Vara del Rey, Madrid, 2011” [33] in which public spaces are regenerated through intelligent trees that create shaded spaces and capture solar energy. Also noteworthy from this architectural firm is the “Urban Tree” project [34], which builds a meeting point for exercise. Its canopy is configured by a set of photovoltaic solar panels and green panels with aromatic plants. The base has resting points and bicycles that activate the tree.

The Andrés Jaque/Office for Political Innovation architectural firm also built in 2015 the “Cosmo” [35] prototype in New York, an artifact designed as a pleasant and climatically comfortable garden whose main mission is to make water drinkable. Through the COSMO app, it is possible to follow the evolution of the water in the device, to learn the insight needed to construct similar devices, and to connect with the community of experts that participated in its design.

Also approaching the scale of the Smart Tree, a small-scale industrialized object that improves the comfort of its immediate environment and shares information from its sensors, we currently find projects that partially develop some of these objectives, such as the project of vertical green panels with integrated benches from the start-up Green City Solutions whose prototype has been installed in some countries such as France, Germany, Belgium or Hong Kong [36]. Or the student-run Northwestern US Private University Illinois State Industrial Design Project to build a solar-powered charging station. Or also the Smart Palm Prototype in Dubai from the company D Idea Media for the creation of an artificial palm tree with WiFi connection [37].

However, unlike the aforementioned projects, Smart tree is designed using recycled elements. The restructuring of this existing structure into a new pavilion involves a minimal investment in both, material and energy resources. A transformation strategy that reduces the consumption of resources to practically zero in construction, which account for about fifty percent of those generated by the planet, making it one of the least sustainable industries [38]. This consumption of resources not only occurs in the construction of new construction, but also in the rehabilitation of buildings, since the waste from demolition and construction activities is one of the largest consumers of resources produced in Europe [39].

Smart Tree responds to the reduction of these handling processes, giving a second life to construction elements with minimal or no transformation [40]. In response to the need to minimize the exploitation of resources, it is necessary to develop strategies for a sustainable development not only of new buildings, but it is also necessary to raise the problem of sustainability in the rehabilitation of existing buildings. The main reason for the obsolescence of buildings is not usually due to the fact that their bearing structure has reached the end of its useful life, but to the new functions and needs that society requires [41]. Consequently, extending the use of buildings reverts to the generation of less waste and, consequently, to the reduction of carbon emissions.

The urban greening of an area where the technology is the protagonist enables it to be used by humans as a pleasant and different place from the immediate environment, due to the climatic conditions softened by the urban greening with plants, especially in Mediterranean areas with a very hot and dry summer season [42]. In the Mediterranean area where our project is developed it is important to consider the existence of heat stress in outdoor environment under arid climatic situations [43].

The human body’s physiological responses to the environment integrate various physical phenomena that interact with the space (light, noise, vibration, temperature, humidity, etc.) [44] which can be also capable of being monitored by sensors due to the development of a new knowledge in green information and communication technologies (ICT) [45,46], which report the improvement of the climatic temperature, humidity and CO_2_ parameters within the technological tree, leading to the local mitigation of the global problem.

Thermal comfort is defined by [47] as “that condition of mind that expresses satisfaction with the thermal environment and is assessed by subjective evaluation”. So, this comfort is in relationship with the relative humidity and wind speed in the environment.

In addition, regional floras can constitute the best database with information to select the species with which to achieve the effects of nature, especially based on native taxa adapted to local Mediterranean seasonally climatic conditions and with a variety of functional types, which will have their role for different purposes [48].

In terms of ICT, Smart Campuses are attracting a growing level of interest by the research community. Min-Allah & Alrashed in [49] sketch the main principles of the Smart Campus paradigm. Multiples applications of ICT are described, emphasizing the concept of “microgrid” as a system able to produce electricity and being able to connect and disconnect from the general main grid. Such grids are also linked to multiples services such as recycling, air quality, smart infrastructure, etc.

The work in [50] also consolidates on the services and technologies for Smart Campus, with a general framework supported by an infrastructure of sensors, processors, communications, storage and people. The work also identifies multiple smart campuses around the world, such as in Korea [51], from Lancaster University (UK) [52] and the one from University of Málaga (UMA) [25]. Reference [53] establishes a list of 74 Key Performance Indicators (KPIs) for smart campuses and microgrids and also spotting other smart campus initiatives.

From the University of Málaga, the work in [25] establishes how the UMA Smart Campus wide ICT infrastructure combines multiple sensoring and communication layers as well as multiple educative and research activities under the same general framework. The new Smart Tree system to be defined in the next sections is a completely new element which follows the concept of microgrid fitting into UMA general infrastructure.

## 3. Context, Requirements and Proposed System

In this scenario, the University of Málaga approved in 2019 the Smart-Campus initiative [25] with the aim of supporting projects in the field of sustainability and Smart Cities through collaboration in interdisciplinary working groups composed of teachers and students.

Within this framework of sustainability and cooperation, the proposal of Smart Tree project was born. In it, the general objective was defined as the creation of a space for vegetation, work and leisure, made available to the community and providing clean energy to its users, as well as gathering environmental data acquired through an IoT system.

**Figure 1:**
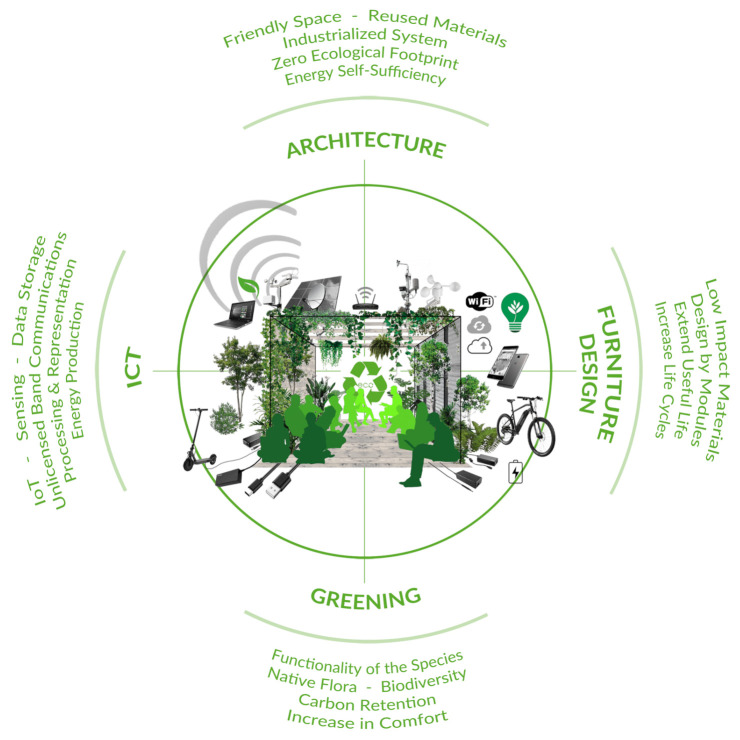
Smart tree multidisciplinary concept scheme.

In this way, the Smart Tree was designed as an experimental prototype where four disciplines participate as shown in Figure 1: architecture, furniture design (with a special focus on circular economy), greening and ICT.

At the university level, the Smart Tree experience aims to create a small-scale experimental laboratory of the systems to be applied in smart cities. The necessary conditions are created for that architecture under sustainable criteria [54] where nature and new technologies are combined in symbiosis to improve comfort conditions.

The proposed environment is based on 7 of the 10R principles [55], such as Reformulate, Reduce, Reuse, Remanufacture, Recycle, Revalue, Redesign. Smart Tree, “reformulates” the process of rehabilitation and detection of elements with possible second life; “reduces” the new materials to be incorporated and minimizes the manipulation of the elements; “reuses” all the materials in a new configuration reducing the incorporation of new materials; “remanufacture” through a low-impact industrialized assembly process; “recycles” elements with minimal transformation of the original material; “revalues” with renewable energies, greening of the environment and communication and information technologies on environmental parameters; “redesigns” a new object with new functions from the efficient reuse of a priori elements destined for disposal, imitating the natural ecosystems so that each part becomes the next link in the chain.

Specific guidelines and requirements are in this way identified for each of these integrated disciplines.

### 3.1. Architecture

Smart Tree’s architectural design goals are based on the following concepts:

1. Generation of friendly spaces for leisure and study surrounded by greenery.
2. Inventory of materials and construction elements from the UMA wastes destined for disposal.
3. Reused materials: A second life cycle that, through a new architectural ecodesign, provides a new use in a new context.
4. Industrialized architecture: Industrialization understood as architectural production method and sustainable construction system that can respond to needs of workspaces and housing in large cities [56]. At the same time we consider dry assembly systems as a tool that allows the incorporation of reused materials into the construction process [57], and also makes it possible to recover and recycle the materials after their life cycle, opening the way to the so desired building with ecological footprint 0.
5. Transformable and Adaptable to spaces both outside and inside public buildings.
6. Activating space of nodes or green islands: once the vegetation grows, the prototype shall be able to be transported and migrate to other places to green them and provide it with ICT systems.
7. Scalability to each specific place and location.
8. Natural vegetation support structure.
9. Urban furniture support (ergonomic and ecodesign) for indoor and outdoor living and working rooms.
10. Energy self-sufficiency: Support of photovoltaic collectors included a storage system back-up.
11. Support of a network of environmental and subsoil sensors.

### 3.2. Cyclic Economy

From the recycling point of view in particular and to reduce the negative effects on the environment, environmental criteria should be included in the design process [7]. Ecodesign fits this premise as it is defined in UNE-EN-ISO 14006:2020, “as a systematic approach, which considers environmental aspects in design and development with the aim of reducing environmental impacts during the life cycle of a product”. In ecodesign, the life cycle (LC) concept is principal [58,59], as it considers environmental aspects at all stages of the life cycle, from the acquisition of raw materials or their generation from natural resources to the final disposal.

One of the objectives of ecodesign of the Smart Tree is to reduce the use of resources and increase their useful life, using different strategies: recycling materials, reusing or renewing products, reconditioning or remanufacturing, to increase production efficiency [60]. These tools are what the European Commission underlines in its Circular Economy Action Plan [61], to modernize the European economy towards a path of sustainable competitiveness and with benefit for the environment and the economy [62].

The circular economy is one that has a restorative design and aims to keep products, components and materials at their maximum utility and value, at all times [63]. Circular economy is a condition for sustainability [64], the use of waste as a resource, allows us to extract the maximum value from them, allowing them to remain part of the productive fabric. Replacing the use of raw materials with secondary materials (materials that have finished their first life) is necessary to avoid overexploitation of resources and reduce greenhouse gas emissions [65].

For the design of the Smart Tree we used the ecodesign strategies called “design for product life extension” and “design for appropriate end-of-life” [60], both associated with waste management. In this way, the design objectives were:

- Select low-impact materials, prioritizing secondary raw materials.
- Minimize the consumption of auxiliary materials.
- Minimize energy consumption in production, reducing the manufacturing stages.
- Extend the useful life of the furniture, define processes and maintenance stages.
- Optimize the useful life of the product by increasing the number of life cycles.
- Design furniture by modules to facilitate disassembly/assembly.

### 3.3. Greening

In order to create the Smart Tree as an hybrid artificial and natural space and furthermore provide it with native plants that generate a new environmental and sensory microclimate, the greening of the environment was based on the following guidelines:

1. Use of primarily native flora adapted to the Mediterranean macrobioclimate of the environment following the main flora of our Mediterranean area [48].
2. Use of plants of reduced water requirements and maintenance [66]. The use of perennial plants, according with the established concept by [67], was a priority. Reference [48] was the main source of information for this selection, while references [19,42,68] were to provide the information about the requirements of the selected plants.
  From an ecological and functional point of view, these kinds of plants are considered arid-active or persistent species maintaining active shoots during the unfavorable season (which is consider the summer in a Mediterranean area [69]).
3. Use of plants which maintain their leaves all year (evergreen species) in order to provide shade, barrier effect and weather pollution protection to the space through all the year. These plants are to be combined with deciduous plants for areas where the priority is the exposure to sunlight in winter.
4. Use of plants with different timing of flowering in order to create an area with the presence of a range of colors and aroma during all the year [48].
5. Use of plants considering the functionality of the species taking into account the following functional traits [70]: life form, plant height, growth form, crown diameter, canopy density, type of fruit and seasonality. In addition, we considered effective plants at reducing air pollution (traffic emissions) through the leaves fixation of particles [71,72,73,74].
6. Finally, we avoid using toxic plants by consulting [75] in order to make safe the natural part of the Smart Tree space.

### 3.4. ICT

In terms of the ICT infrastructure, this represents a key factor in the Smart Tree architecture. A key of its objective is to measure different environmental variables in order to provide regular data on their values over time in an easy way to process and be used for the maintenance of the Smart Tree structure, furniture and greening as well as to support associated research by the university community.

This translates into multiple sub-requirements:

1. Sensed variables: The system must measure those variables associated to the users’ well-being, the vegetation growth and management as well as those associated with the impact of the infrastructure in its environment.
2. Communications: All communications should use unlicensed bands and avoid operational costs (e.g., telecommunications subscriber fees).
3. Avoid interference: The general WiFi network (Eduroam [76]) of the campus should be used, without creating additional purposely deployed networks. This requirements has the objective of not generating an excessive number of WiFi networks and interference in the campus and, at the same time, allowing the different nodes to be registered and identified as a group. However, it will add a layer of complexity to the ICT system due to the limitations of micro-controllers to comply with the certificates and security mechanisms associated to such as networks [76].
4. Data storage and reception: The system must be able to receive data in an efficient manner and in turn store it securely in a database.
5. Monitoring and alerts: In order to know the status of Smart Tree, it was necessary to have an intuitive and user-friendly interface where all the received data is displayed. In order to be able to have relevant information in real time without the need for periodic revisions to the above interface, it was necessary to have a system with the ability to make alerts at times when human intervention is considered relevant.
6. Flexibility: For the sake of future improvements and changes, it is vital to implement a versatile system that is as modular as possible.
7. Energy efficiency: The system must be powered with sustainable energy generated by the infrastructure, pursuing the lowest energy consumption possible.
8. Portability: the ICT architecture shall also allow the general dismantling and mounting of the general architecture and the possibility to connect or not to the general electric and communications grids.
9. Education: Given the nature of the university as a teaching institution, one key objective of the ICT platform is to serve as a way to train and educate engineering students in the development of IoT systems. Therefore, open/do-it-yourself equipment will be prioritize over off-the-shelf solutions.

Considering all these requirements for the different fields of the Smart Tree, the following sections present the main design focusing on each of its main principles: architecture, furniture, greening and ICT.

## 4. Architecture

The architectural design of the Smart Tree environment is designed as an scalable and exportable system with the power to transform its immediate environment through the greening of both the architectural object, a “Folie” in a garden, and the specific place of location creating a friendly and healthy environment both physically and psychologically [77].

At the beginning of the investigation, a waste map of the University of Málaga was made, from which materials were glimpsed that through minimal manipulation or transformation could be “reused” in the structure and envelope of the “Smart Tree” prototype and in the integrated urban furniture.

**Figure 2:**
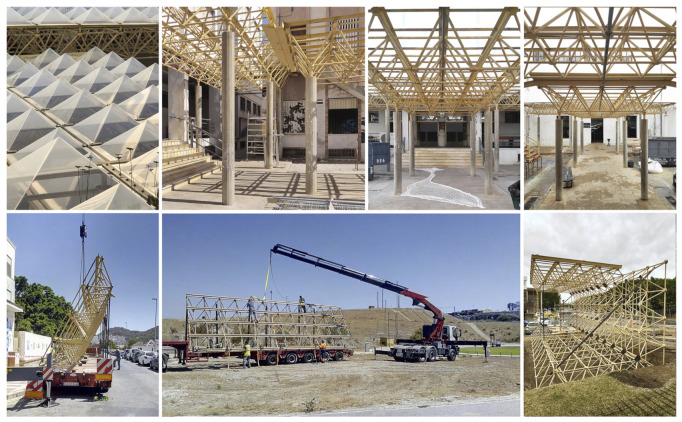
Images of the construction process of the Smart Tree. The top row shows the previous state of the structures located in the courtyard of the Faculty of Fine Arts. The bottom row shows the process of moving and assembling the recycled structures next to the Faculty of Science.

In this way the system is designed from the reuse of a identified structure that served as a “pergola” in the courtyard of the Faculty of Fine Arts which was destined for recycling as a result of the necessary rehabilitation of the building. This original structure and its evolution towards the Smart Tree structure can be seen in Figure 2.

From this material, three triangulated spatial structures top row in their original status were obtained that served to compose the pavilion with minimal manipulation and transformation operations. We also reuse the methacrylate domes on the roof and the concrete columns as foundation piles. The floor covering was made with cross-laminated wood from concrete formwork panels.

**Figure 3:**
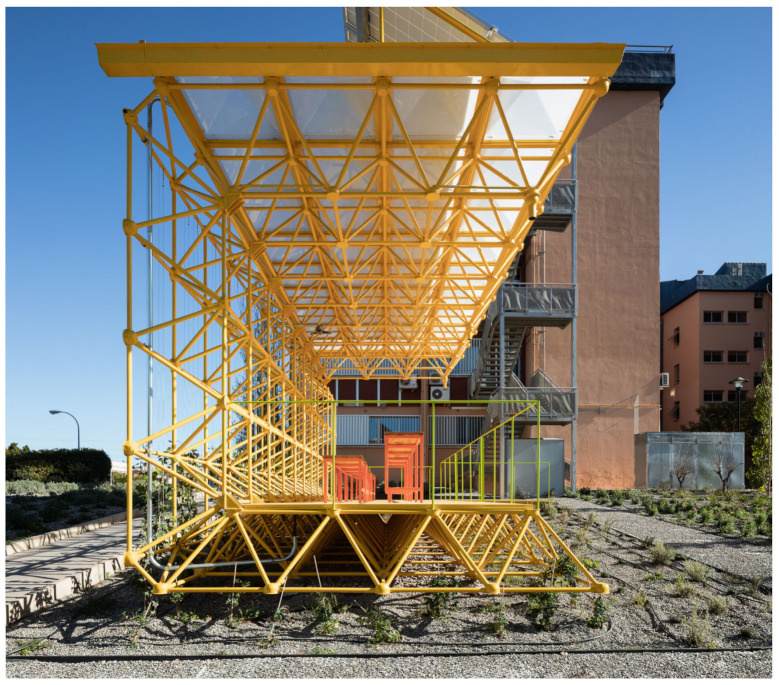
Structural order of the Smart Tree architecture.

**Figure 4:**
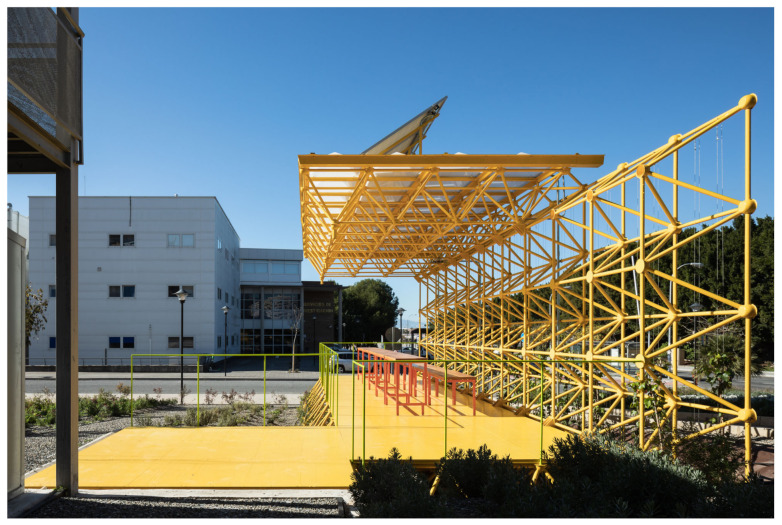
View of the Smart Tree area from the entrance.

The first of these three triangulated spatial structures were used to build the ground plane that raises the pavilion above the garden and the second as a vertical wall that protects users from the noise of the road to the north. This second structure supports the third, which is formed as a cantilevered roof that generates shade and protects users from the sun and rain. The new order of placement of the structures can be seen in Figure 3 and Figure 4.

These show how the pavilion rises above the garden, situated between a road to the north and west (Figure 4 back and right) and an opening up to the south (Figure 4 left). Under the covered area is the recycled furniture and the photovoltaic panels for collecting solar energy are installed on the structure.

**Figure 5:**
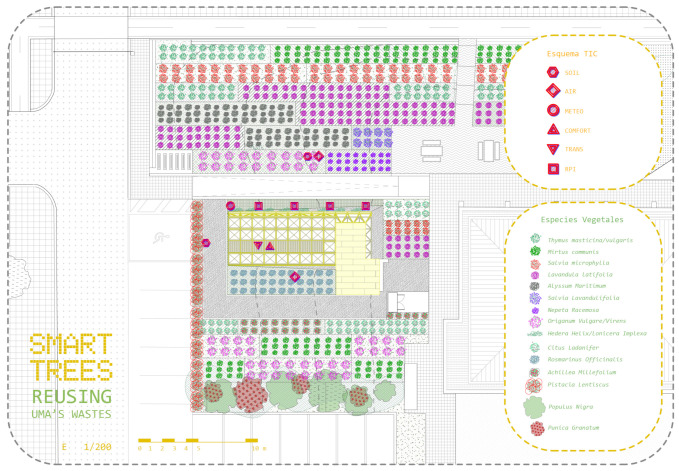
Situation map of the Smart Tree area, vegetation an location of the sensors and ICT elements.

As shown in Figure 5, the resulting Smart Tree is an open-air classroom-pavilion of 48 m^2^, of which 36 m^2^ are covered with a methacrylate roof and 12 m^2^ are uncovered. The intervention proposes greening an area of 522 m^2^ in its immediate surroundings. The pavilion is located in the central part of the area, protected by strips of vegetation from noise pollution from the road to the north and from the car park to the south and west. A pedestrian path crosses the garden in the central area connecting Smart Tree with the boulevard that serves as the main communication axis of the Uma university campus. The different species of plants and trees used and the location of the sensors are depicted, which form Smart Tree as an inseparable ensemble of architecture, botany and new technologies.

**Figure 6:**
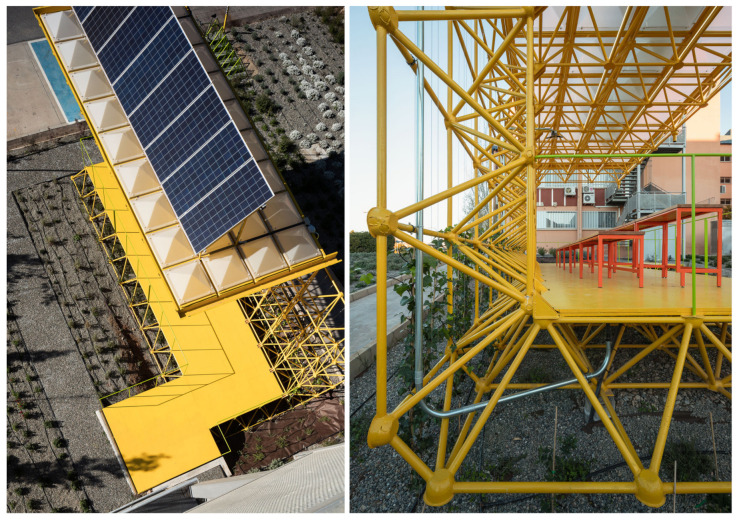
(**Left**) photovoltaic panels attached to the pavilion structure. (**Right**) the sensor connection ducts attached to the pavilion structure.

Figure 6 shows how the resulting structure allows to be colonized by vegetation, supports photovoltaic panels for energy self-sufficiency, and at the same time can contain lighting lines, and the electricity and ICT network.

### Cyclic Economy Strategy and Furniture Design

The design strategies established to define the furniture in the Smart Tree were developed using an ecodesign methodology to schedule the necessary time and resources. The ecodesign methodology does not change the structure in the traditional design of a product, it only adds environmental specifications along its LC. For the systematization of the design, the following stages were carried out: Project preparation, Analysis of Environmental Impacts, Generation of ideas, Development of concepts, Product in detail, Action Plan and Evaluation [78].

For the generation of ideas stage, the tool designed by C. van Hemel (Lids Wheel) was used, which defines eight phases that helped the team to approach a sustainable design of the Smart Tree in its LC [79]. In addition, these phases consider the 10 R’s, i.e., the circularity strategies defined by the EU: Recover-Recycle-Reuse-Remanufacture-Refurbish-Remelt-Renew-Repair-Reuse-Reduce-Reformulate-Rethink-Refuse. These circularity strategies reduce resource consumption and environmental pressure by increasingly minimizing the amount of primary products and materials needed [62]. Grouping both parameters, the following phases have been performed for the generation of ideas for furniture:

Following this, a cataloging of discarded products from the various facilities of the University of Málaga was carried out, as well as visits to university buildings that were in the process of remodeling. After this, the products were cataloged according to their material, use and state of conservation. The most common products were benches, countertops, wood and metal shelves, other wood and metal structures, desks and tubular profiles.

The production techniques were done at the UMA workshop, where the processes to obtain the new product were: polishing, cutting, water painting and assembly. The designed furniture does not require energy input for its operation, its maintenance is based on cleaning twice a week and every year it will be painted to preserve its attractiveness and ensure its functionality. Once its second useful life is over, as it can be disassembled, each element can be evaluated to reintegrate them into the production system, recycle them, or take them to the appropriate waste manager.

**Figure 7:**
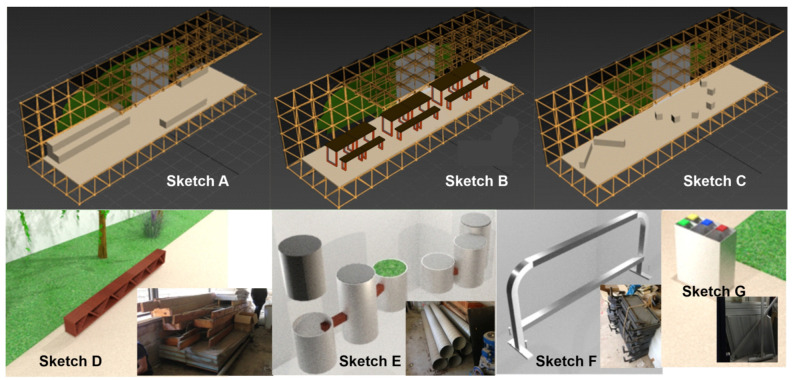
Sketches of furniture for Smart-Tree: (**A**) structure with wooden benches; (**B**) structure with tables and workbenches; (**C**) round table version with wooden cubes, plus annex of rectangular benches; (**D**) bench implemented with wooden beams; (**E**) bench with metal tubes and concrete; (**F**) bicycle rack with handrails; (**G**) recycling container with metal shelves.

Different furniture concepts were developed with these premises that evolved with the Smart-Tree architecture and the stored products (see Figure 7). A prioritization matrix was used based on environmental improvement, design criteria, technical criteria, economic criteria and according to the motivating factors of the project.

**Figure 8:**
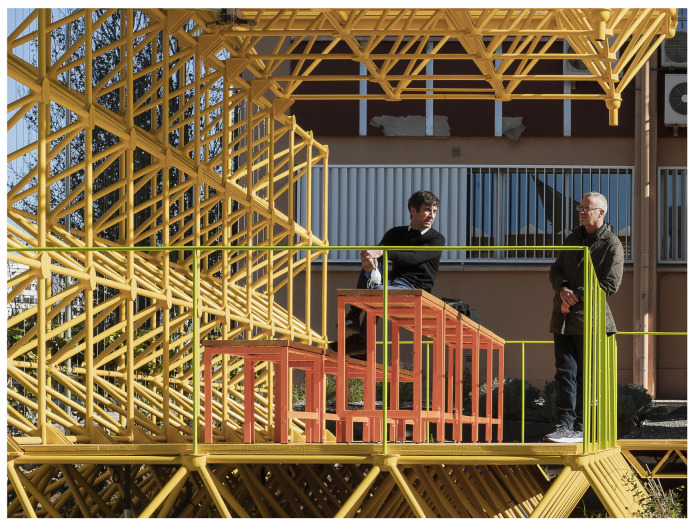
Smart Tree furniture.

The Sketch B shown in Figure 7 was the implemented design (as shown in Figure 8), consisting of a set of five subsets of benches and tables. Both the metal structure and the top of the benches and tables were obtained from a laboratory of the Faculty of Sciences of the UMA that was being remodeled (Rethink-Reuse-Reduce-Reduce-Reduce). After a cleaning and polishing treatment, both elements were painted, the metal structure with orange oil-based paint and the worktops with water-based varnish (Re-Fabricate-Reduce-Restore-Recycle).

It was decided to monitor the state of conservation of the Smart Tree furniture every 6 months, as well as to update the catalog of products discarded by the different UMA centers.

An environmental evaluation of the design was carried out. To achieve this, the Environmental Aspect (AA) of the “Laboratory Table of the Faculty of Sciences” was analyzed, comparing two different scenarios: Scenario A, the table, once the laboratory is remodeled, becomes a waste and therefore goes to a landfill (Mesa Lab); and Scenario B, where the lab table finishes its first LC and is reused to start its second LC (Smart Tree Table).

**Figure 9:**
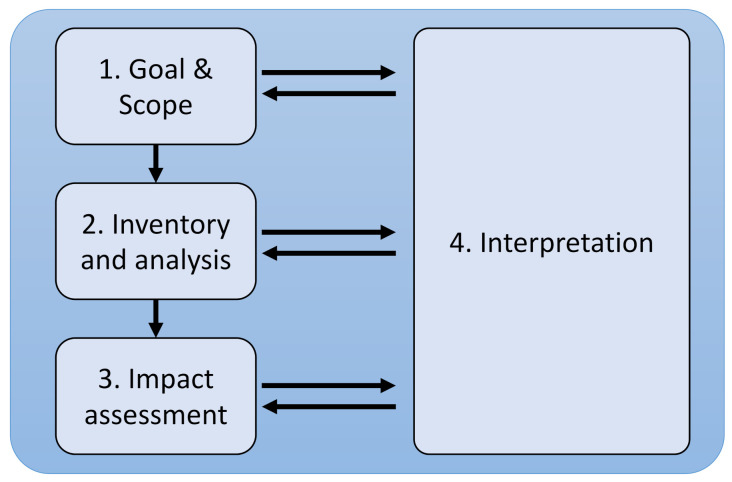
Framework LCA—ISO 14040.

A simplified Life Cycle Assessment (LCA) of the product was performed following ISO14040 [80,81], the methodology ReCiPe Endpoint 1.06 [82] and the database (DB) Ecoinvent 3 [83]. In this way, the LCA is carried out in the four phases (as represented in Figure 9) defined by the standard. The first phase of goal and scope definition consists of establishing the objectives and scope of the study (which is an important step, especially for comparative analysis), the system to be studied and its boundaries, the quality of the data, the assumptions and the level to be reached. The functional unit (FU) is also defined in order to be able to associate the inputs and outputs of the system. Secondly, in the inventory phase, data are collected and quantified to determine the inputs and outputs of matter and energy of the system, including the use of resources (raw materials, energy, water, etc.), and the emissions to air, water and soil that are produced. In our analysis, this data is obtained via computational transformation using baseline values coming from the state of the art as well as from the field study in the UMA workshops. Thirdly, the impact assessment stage is a technical process aimed at understanding and evaluating the magnitude and significance of the potential environmental impacts of a system throughout its life cycle. The assessment is carried out in stages, which are differentiated between mandatory and optional. Finally, there is the stage of interpretation of results that provide information consistent with the defined objective and scope, leading to conclusions and recommendations in decision-making for the recipient.

**Figure 10:**
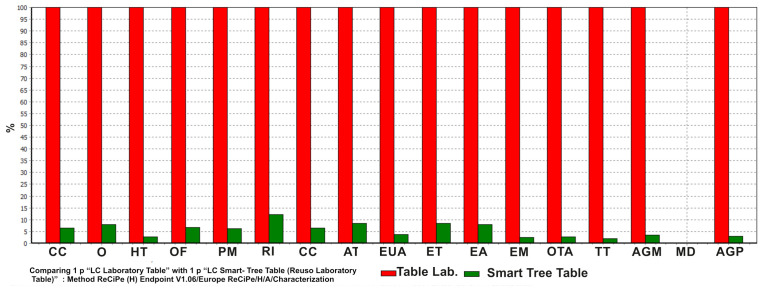
Comparative LCA between furniture-Characterization.

For the realization of the Life Cycle Inventory (LCI) [84,85] the Impact Categories included in this study were [83]: Climate Change (CC); Ozone Depletion (O); Human Toxicity (HT); Photochemical Oxidant (OF); Particulate Matter (PM); Ionizing Radiation (IR); Climate Change. Ecosystems (CCE); Terrestrial Acidification (AT); Freshwater Eutrophication (EUA); Terrestrial Ecotoxicity (ET); Freshwater Ecotoxicity (EA); Marine Ecotoxicity (EM); Occupation of Agricultural Land (OTA); Land Transformation (TT); Metal Depletion (AGM); Fossil Depletion (AGP). The results are shown in Figure 10), where it can be observed that the IA in all categories of the “Smart Tree Table” improves by more than 90%.

**Figure 11:**
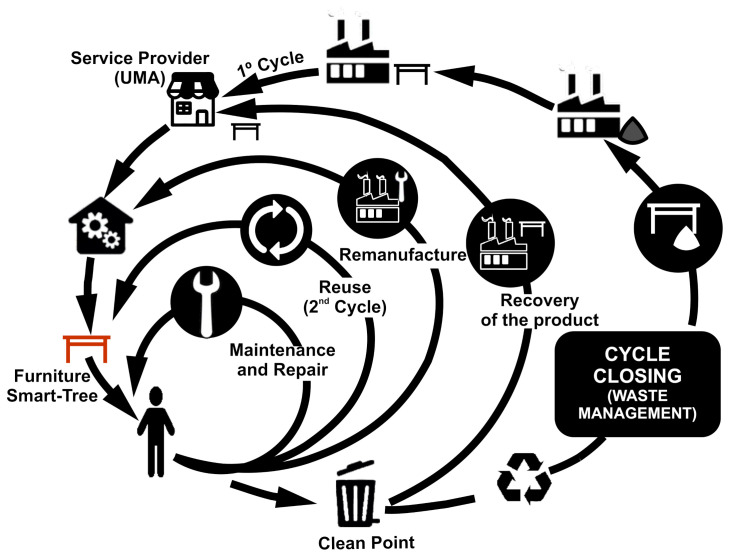
Smart-Tee Table LC Circularity Rings.

Finally, the circularity strategies of the Circular Economy of the Smart-Tree furniture were analyzed. Following the work of Fernández Alcalá [60], a rings graph on the LC circularity of the Smart Tree was generated as shown in Figure 11, showing that the starting assumptions have been fulfilled.

## 5. Greening

**Figure 12:**
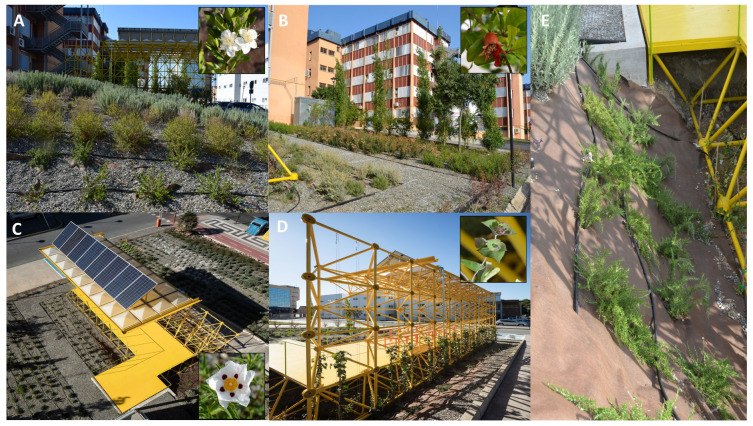
(**A**) Northern green wall constituted by the evergreen shrub *Myrtus communis* with a detail of the flower. (**B**) Southern green wall constituted by the combination of deciduous species *Punica granatum* and the tall tree *Populus nigra* with a detail of a fruit. (**C**) Bands showing some of the mediterranean-type xerophytic scrubs with a detail of a *Cistus* flower. (**D**) Northern green wall showing the vertical greened of the northern metallic tubular structure with *Lonicera* and *Hedera* species with a detail of flowering buds. (**E**) Prostrate scrubs of *Rosmarinus officinalis* var. *postratus* covering the bottom and ground part of the structure.

Taking into account the aspects provided in Section 3.2 and through the use of ArcGIS software (GIS software) version 10.4.1, the design of the most optimal greened was developed organising it in six main areas as shown in Figure 12A.

1. A wide northern green wall was created using an evergreen native plant of reduced water requirement *Myrtus communis* (*Myrtaceae*). This species was used as barrier because it is an evergreen scrub species of short height.
2. A western green wall was made using the sclerophyllous evergreen *Pistacia lentiscus* (*Anacardiaceae*) in order to filter the pollution produced by the circulation of vehicles, since it is considered an effective plant at reducing air pollution (traffic emissions). This species is a plant of reduced water requirement and maintenance that can be pruned. In addition, their aerial parts (leaves, twigs and berries) present essential oils [86] that could dissimulate the pollution.
3. A large southern green wall was generated using the combination of deciduous species such as the tall shrub *Punica granatum* (*Punicaceae*) and the tall tree *Populus nigra* (*Salicaceae*) which present mixed yellow and red colors during autumn and absence of leaves during winter, period when the sunlight can penetrate to the Smart-Tree. Moreover, the fleshy fruits of the *Punica* species can provide nutrients to the urban avifauna as another environmental contribution. This barrier provides shade during summer (when the plants show green leaves) but allow the exposure to sunlight in winter (leaves dropping takes place in autumn) (Figure 12B).
4. The rest of the area was covered using different bands of Mediterranean scrubs, which provide aroma and color throughout the year, recreating a sort of natural plant ecosystem typical of Mediterranean areas, the following species were used (Figure 12C): *Thymus mastichina* (*Labiatae*), *Salvia lavandulifolia* (*Labiatae*), *Alyssum maritimum* (*Brassicaceae*), *Achillea millefolium (Asteraceae*), *Cistus ladanifer* (*Cistaceae*) and *Origanum vulgare (Labiatae*), among other plants. Most of these plants are nectar and pollen producing species for honeybees and other pollinators.
5. The northern metallic tubular structure was covered using climbing plants from a functional point of view: a combination of *Hedera helix (Araliaceae*) and *Lonicera etrusca* (*Caprifoliaceae*) was used (Figure 12D). This way it acts as a barrier for noise and pollution from the main boulevard of the area.
6. On the ground close to the bottom of the Smart Tree: *Rosmarinus officinalis* var. *postratus (Labiatae*) was used in order to cover the structure in its bottom part (Figure 12E).

## 6. Sensing and Communications Approach

ICT, greening and architecture and closely linked in the Smart Tree, as a proper sensing infrastructure is required to both monitor the status of the environment variables impacting the vegetation as well as to measure the impact of both architecture and greening in the general conditions experienced by the user of the infrastructure.

In this way, the ICT technologies included in the project are divided in three main areas. Firstly, the sensors used to gather information from the environment and greening. Secondly, the communications required to gather these measurements. Thirdly, the representation and analysis tools used to make the data available and useful for the management of the environment and research.

### 6.1. Sensors

In order to obtain the variety of measurements specified in Table 1, different types of parameters are to be measured in the Smart Tree to gather its status and impact in the environment. Here, the monitoring focuses on key variables affecting both the users of the infrastructure and the vegetation of the area. For the users, the parameters of interest are those affecting their comfort and well-being. For the vegetation, air, weather and soil variables are to be considered.

Two general measurement areas “exterior” and “interior” can be identified, for those variables measured outside and inside the structure, respectively. Considering this, the external variables include the air temperature (°C), wind speed (m/s), wind direction (degrees), CO_2_ concentration (ppm), illuminance (lux), air atmospheric pressure (hPa), air humidity (%) and rain (mm).

The internal variables are the air temperature (°C), air humidity (%), air atmospheric pressure (hPa) and illuminance (lux). These variables are measured also in the exterior allowing for the comparison between both and the assessment of the impact of the Smart Tree structure.

Following the guidelines established in Section 3, a key aspect of the design has been selecting equipment that allow engineering students to learn about sensing, programming and telecommunications, having as their role to assembly the different parts of the system and validate it. Therefore, “do-it-yourself” (DIY) solutions has been prioritized, defining sensing nodes form by the integration of individual sensors connected to the microcontroller needed to gather and transmit their data. As the cornerstone of this design the SparkFun ESP32 Thing microcontroller has been selected due to its versatility, low-cost and common use as a teaching platform [87].

The specific sensors for each node-type can be found in Table 1, with indications on the type of node that they belong to and the communication protocol they use to connect with the ESP32. Some of these sensors have been selected because they present some useful features for the project other than the previously mentioned DIY focus. Hence, the temperature and humidity sensors (DS18B20 and SKU-SEN0193 respectively) have a probe encapsulation format that easily allows their introduction into the soil, as well as presenting a good accuracy in the range of expected temperatures. The humidity sensor also has a relevant feature compared to other DIY sensors of the same category, particularly its capacitive nature [88], instead of resistive, which allows for a longer lifetime due to its insulation and consequent reduced exposure to corrosion [89]. In the case of the CO_2_ measurement, the majority of the gas sensors of the DIY market are analogic electrochemical sensors that require pre-heating and whose measures tend to drift strongly with time. Conversely, the selected SCD30 component is NDIR (non-dispersive infrared) sensor. This works by the emission of infrared light through a portion of air. Depending on the quantity of CO_2_, a certain amount of this light is absorbed, providing accurate measurements [90]. It also includes an internal temperature sensor that allows the automatic correction of the measurement.

From this, and linked with the greening and environmental needs, four types of sensing nodes were established with different sets of variables to measure. The SOIL node provides data about the temperature and humidity of the terrain of the environmental inner and outer Smart Tree conditions. The AIR node gives data about the CO_2_ concentration in the air which is monitored because of being the principal chemical causing global warming and the general atmospheric temperature and humidity of the environmental inner and outer Smart Tree conditions, since they are needed to make corrections on the CO_2_ concentration measurement and increment its accuracy.

The COMFORT node is in charge of providing environmental data like the air temperature, humidity, atmospheric pressure and illuminance. Finally the METEO station is the node with the largest amount of sensors, air temperature, humidity, atmospheric pressure, illuminance, air quality, rainfall, wind speed and wind direction.

**Figure 13:**
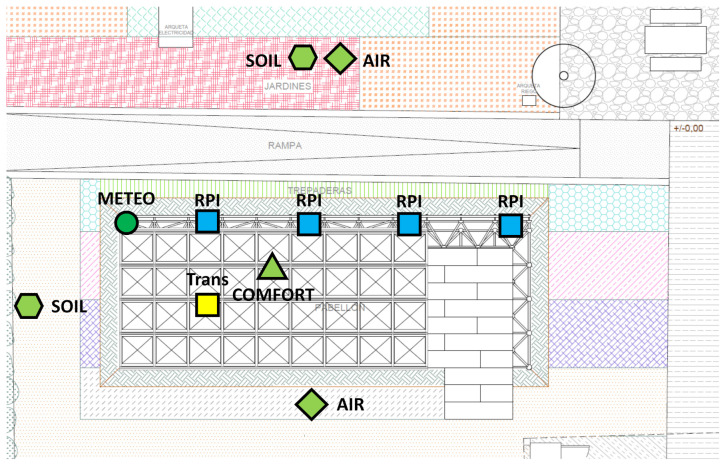
Diagram of ICT elements positioning. Sensing nodes (green), central nodes (blue) and electricity supply connection (yellow).

As shown in Figure 13, the nodes have been distributed over the structure and the terrain in order to be able to measure the environmental conditions provided by the Smart Tree (COMFORT), compare them with the status outside it (AIR, METEO) and to provide data to support the greening monitoring (SOIL, AIR, METEO).

The link between this design and the greening of the Smart Tree is shown in the Table 2, where each of the selected plant areas were associated with a different sensing node. In this way, the SOIL nodes monitor the environmental conditions of the plants providing data of the temperature and of the levels of humidity of the soil in order to augment the efficiency of our irrigation system. Moreover, the sensors of air temperature and soil humidity, provide parallel related data for the seasonal flowering and growth phenology of the bands of scrub plants [91]. In addition, the main area of the Smart Tree has been monitored with a specialized node (COMFORT node) to measure the environmental variables in relationship with human comfort according with [43,92] (thermal comfort and heat stress).

### 6.2. Communications

**Figure 14:**
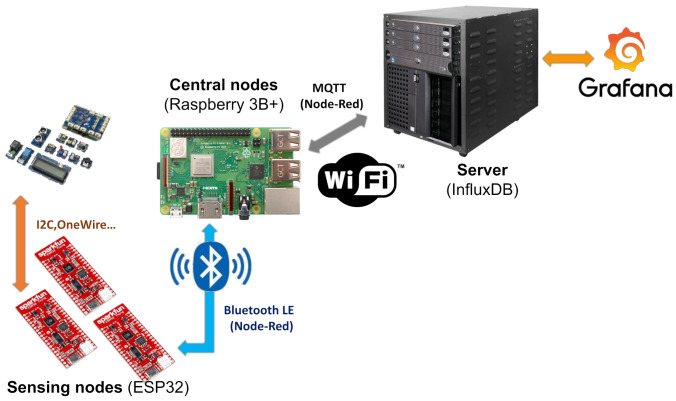
Data path diagram and communication technologies.

After the gathering of the data by the sensing nodes, the information shall be transmitted for its analysis and representation. In this way, Figure 14 represents the path that the information follows from its collection by the sensors to its presentation in the server’s graphical interface.

Communications and data collection is done through a capillary architecture going starting from the already mentioned sensing nodes based on SparkFun ESP32 Thing, distributed in a suitable way to perform the measurement at the points of interest. It was deemed necessary to reduce energy consumption and avoid interference with the pre-existent WiFi Eduroam by not deploying new WiFi networks. This, as well as the the limitations of the microcontroller to connect to Eduroam due to its limitation to follow the security restrictions of such as networks and the limited coverage of the area, led to the use of Bluetooth Low Energy (BLE) instead. In this way, intermediate concentrators (“central nodes”) were based on Raspberry Pi 3B+ being responsible for receiving the data from the sensing nodes via BLE and sending it through the Eduroam WiFi connection to the university network and to the project server.

These central nodes were distributed over the structure and as shown in Figure 13, which shows the sensing nodes in green and the central ones in blue, where the BLE connection has an approximate reach of 25 m.

Knowing the system infrastructure and the connections between the sensing and central nodes, the data collection was defined as different state machines implemented by the sensing nodes (sensor boards) and the central nodes (Raspberries/gateways).

In this way, the sensing nodes run their program, starting a BLE “server”, and taking measurements (through the different interfaces such as I2C, OneWire…) offering the last acquired data to the central nodes, until they receive it and indicate the peripheral nodes to enter sleep mode by means of a sleep signal.

To do this, the central nodes must control the timing and synchronize the sensing nodes for a correct data flow in time. In simplified form, when a central node starts up, it searches for available BLE servers from which to take data, when it finds them, it takes the data in a given time, and after this, it signals all the connected peripherals to enter sleep mode for a given time until the next shift. To do so, it uses various counters and control variables, while indicating to the nodes the entry into sleep mode. At last, it formats the obtained data and sends them via MQTT (Message Queuing Telemetry Transport) protocol [93] to the server for storage.

**Figure 15:**
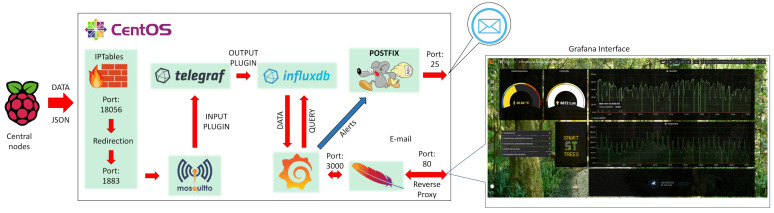
Insight of server software and datapath from reception to graphical representation.

From the server side, an MQTT messaging broker called mosquitto [94] was used as shown in Figure 15, dedicate to receive all the data coming from the central nodes. Once data has been received it needs to be stored for future analysis. InfluxDB was selected for this purpose because as an open-source database platform focused on time series [95].

### 6.3. Representation

The project server functional blocks are represented in Figure 15. Here, once the data is received by mosquito and stored in the InfluxDB database, it is displayed in a user-friendly interface based on the open-source visualization system Grafana. Additional operations are performed on the data in order to detect errors and make them less susceptible to noise such as averaging or analyzing the standard deviation. Regarding its visualization, a reverse proxy was configured in order to display the Grafana interface through port 80 due to security restriction.

On the other hand, an alert system is defined in order to warn the maintenance personnel on configurable alarms (e.g., in case soil humidity goes beyond a predefined threshold for a certain time). For this system, e-mail communication was chosen. To develop this function, Grafana was coordinated with an MTA (Mail Transport Agent) based on Postfix.

## 7. Discussion, Lessons Learned & Open Research Challenges

The provided ICT-based data gathering and representation allows for a detailed analysis of the environmental conditions and the impact of the greening and the architecture. This can be studied as temporal series as well as exported for its posterior processing, such as for statistical analysis.

**Figure 16:**
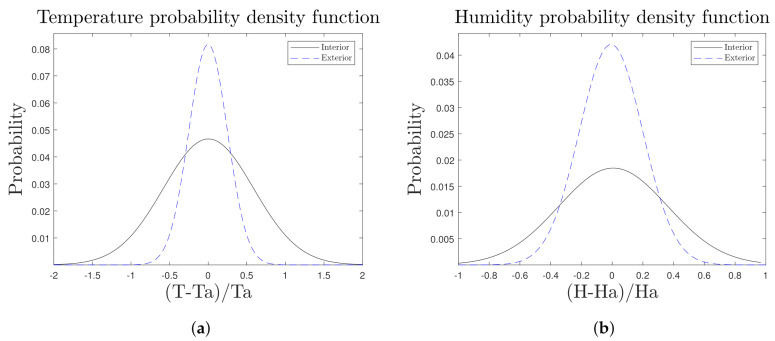
Comparative graphs of temperature and humidity PDFs of the COMFORT (Interior) and METEO (Exterior) nodes, obtained for the month of December. (**a**) Temperature PDF, (**b**) Humidity PDF.

**Figure 17:**
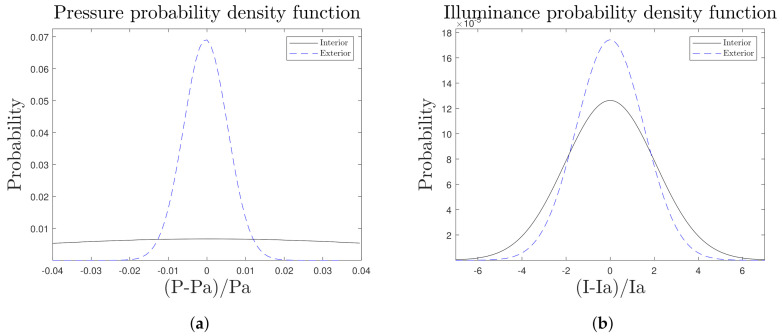
Comparative graphs of pressure and illuminance PDFs of the COMFORT (Interior) and METEO (Exterior) nodes, obtained for the month of December. (**a**) Atmospheric pressure PDF, (**b**) Illuminance PDF.

As a key evaluation of these analysis, the data obtained from the COMFORT node can be compared with the data provided by the METEO node (Figure 16 and Figure 17) in order to know how comfortable our new space of co-working is depending on the actual external climate. During the first months of operation, our nodes showed an average temperature of 5 °C lower (Figure 16a) inside in comparison to the outside area, and higher humidity values (Figure 16b) under the shelter of the structure. This is directly related to the difference in illuminance too (Figure 17b).

The proposed Smart Tree environment has fulfilled the key objectives described in the title of the research. The vision of integrating architecture, greening and ICT systems is materialized in this prototype, which in addition to offering a comfortable space to share ideas, transmits a message of well-being and sustainability. The Smart Tree offers environmental and botanical data while greening the place with native shrub and tree species.

Four of the policy areas identified as priorities by [96] have been addressed by our project: Energy Efficiency of the ICT Sector, Smart Sustainable Cities, Energy Efficient Buildings and Climate Change Management. The Green ICT [45] implemented by this project has supported the architectural construction and improvement of an urban space with greening. In addition, our project is in line with the projects and initiatives that the European Union enhances to make Europe again a green continent and supported by a more sustainable economy [45].

After the pilot design stage of the Smart Tree furniture, it can be seen that the Circular Economy is a link in the tool chain that uses ecodesign to achieve sustainable designs. The eco-design of the Smart Tree furniture has succeeded in conserving and enhancing natural capital by controlling finite stocks and balancing renewable recurrent flows. This design has provided eco-innovation, decreasing IA, increasing product awareness, decreasing costs, making an attractive product, decreasing legal liability and improving the image of the UMA and increasing the motivation of the community (multidisciplinary project). For this design to be reproducible, it is necessary to continue with the cataloging of the products that have finished their first life cycle in the UMA community, even increasing the cataloging of this type of products from other institutions, creating synergies and control tools. In addition, other materials can be recovered for the remanufacturing of other products, since projects are currently being developed at the UMA for the reuse of plastic waste generated at the university itself [97].

Between the lessons learned from the experience it can be highlighted than the integration between greening and architectural design is possible, due to the high variety of functional forms of plants available in Mediterranean environments, which also makes greening in other types of architectural structures very possible. The choice of species has to be very careful, as there are undesirable functions such as toxicity, presence of allergens or attraction of unwanted fauna that have to be avoided or the timing of the flowering has to be take in consideration and in combination with the different plants. The selection of species may also be conditioned by the availability of native versus non-native plants, so it is important to have a good net that supply native plants.

Sensor-based monitoring green ICT has become essential to control both the phenology and vegetative state of the plants and to know their real bioclimatic influence on the architectural design. The functions of pollution, wind and visual barriers will have to be evaluated a posteriori as they require plant growth and visualization in the appropriate climatic season.

The architectural, technological (sensors) and botanical (greening) conjunction has provided good results, as their respective functionalities have been combined and have fitted together correctly so that the interior bioclimate of the Smart Tree depends as much on the artificial structure and the measurement of the sensors as on the selected plants themselves and their exact location.

The combined planning of sensors, the supply of connections, the use of renewable energy, and the development and design of furniture, also recycled, complete the idea of a near Zero Energy Building (nZEB) prototype capable of generating a space with the power of attraction, of transmitting its qualities with objective data and to present a new connective and communicative functionality between students, teachers and the educational community.

Both the maintenance of the greening to ensure that it continues to fulfill its functions (vegetative growth, flowering, comfort) and the opinions of the users regarding the well-being provided by the plants as generators of the green and friendly framework of the Smart Tree stand out as a future challenge. This new monitored area will allow the community of the University of M’alaga to carry out in the future research projects based on the data obtained from the sensors.

## 8. Conclusions & Outlook

A multi-functional green area of more than 300 square meters has been created allowing the use of an space available for the University of Malaga improving the reduced biodiversity of Campus.

The improved environmental conditions created inside in our Smart tree (lower temperature and high humidity) enable the co-working to our students in more comfortable situation in summer, mostly. Moreover, the Smart Tree area promotes the co-working in the current situation of the COVID Pandemic providing a seasonal alternative to working inside of the buildings an safe, as it is in the open-air. In addition, our project can be considered as a first step to proactively contribute the capacity to cope with and adapt to climate change.

The present work has defined a novel approach for smart outdoor environments for leisure, co-working and vegetation in the smart campus, the University of M’alaga Smart Tree. To this objective, a multi-disciplinary approach has been defined, combining innovative architectural, furniture, vegetation and ICT developments. The resulting system has been implemented showing the capabilities of the approach, the positive impact on the environmental conditions of the area and paving the way for future implementations.

Future works will be supported by further projects and educational activities focusing on increasing the number of available sensors in the infrastructure, deepening the analysis of the gathered data and applying machine learning mechanisms to help in the management and evolution of the Smart Tree.

## Figures and Tables

**Table 1:** Sensing nodes for the measurement of the different ambient variables and associated sensors and interfaces.

Node	Sensors	Measurement	Protocol (ESP32)
SOIL	DS18B20	Soil temperature (°C)	OneWire
	SKU-SEN0193	Soil humidity (%)	Analog
AIR	SCD30	CO_2_ concentration (ppm) and ambient temperature (°C) & humidity (%)	I_2_C
COMFORT	BME280	Ambient temperature (°C), humidity (%) & atmosphericpressure (hPa)	I_2_C
	TI-OPT3001	Illuminance (lux)	I_2_C
METEO	SEN-15901	Wind speed (m/s), wind direction (degrees) & rainfall (mm)	
	BME280	Ambient temperature (°C), humidity (%) & atmosphericpressure (hPa)	I_2_C
	CCS811	Air quality (Total Volatile Organic Compounds)	I_2_C
	APDS-9301	Illuminance (lux)	I_2_C

**Table 2:** Selection of plant functional groups: species, environmental function, location in the Smart Tree architectural structure or surrounding enclosure and monitoring of the plants by sensoring nodes.

Location in the Architectural Structure	Plant Groups	Function	Sensor Monitor
Northern green wall	Evergreen shrubs	Acoustic Wall	SOIL
	*Myrtus communis (Myrtaceae)*		AIR
Western green wall	Evergreen shrubs	Pollution barrier	SOIL
	*Pistacia lentiscus (Anacardiaceae)*		
Southern green wall	Winter deciduous trees and shrubs	Visual barrier	AIR
	*Populus nigra (Salicaceae)*	Pollution barrier	
	*Punica granatum (Punicaceae)*	Sunlight seasonal filter	
		Colour seasonally	
		Birds feeding	
Bands (plain soil around)	Mediterranean-type xerophytic scrubs	Ecosystem model	SOIL
	*Lavandula latifolia (Labiatae)*	Aromatic buffer	AIR
	*Salvia lavandulifolia (Labiatae)*	Bees	
	*Alyssum maritimum (Brassicaceae)*	Chromatic (flowers, leafs)	
	*Cistus ladanifer (Cistaceae)*		
	*Origanum vulgare (Labiatae)*		
	*Achillea millefolium (Asteraceae)*		
Vertical greened of the northern metallic tubular structure	Climbers	Acoustic and green wall	SOIL
	*Lonicera etrusca (Caprifoliaceae)*	Wind Wall	AIR
	*Hedera helix (Araliaceae)*	Bioclimatic internal comfort	COMFORT (internal)
Bottom and ground	Prostrate scrubs	Soil and structure covering	SOIL
	*Rosmarinus officinalis* var. *postratus (Labiatae)*		AIR

## Data Availability

Not applicable.
